# Application of Convolutional Long Short-Term Memory Neural Networks to Signals Collected from a Sensor Network for Autonomous Gas Source Localization in Outdoor Environments

**DOI:** 10.3390/s18124484

**Published:** 2018-12-18

**Authors:** Christian Bilgera, Akifumi Yamamoto, Maki Sawano, Haruka Matsukura, Hiroshi Ishida

**Affiliations:** 1 Graduate School of Bio-Applications and Systems Engineering, Tokyo University of Agriculture and Technology, Tokyo 184-8588, Japan; chrisbilgera@gmail.com (C.B.); s184763z@st.go.tuat.ac.jp (A.Y.); s159807u@st.go.tuat.ac.jp (M.S.); h_ishida@cc.tuat.ac.jp (H.I.); 2 Graduate School of Engineering Science, Osaka University, Osaka 560-8531, Japan

**Keywords:** metal oxide gas sensors, sensor networks, gas detection, gas source localization, artificial neural networks, CNN-LSTM, machine learning

## Abstract

Convolutional Long Short-Term Memory Neural Networks (CNN-LSTM) are a variant of recurrent neural networks (RNN) that can extract spatial features in addition to classifying or making predictions from sequential data. In this paper, we analyzed the use of CNN-LSTM for gas source localization (GSL) in outdoor environments using time series data from a gas sensor network and anemometer. CNN-LSTM is used to estimate the location of a gas source despite the challenges created from inconsistent airflow and gas distribution in outdoor environments. To train CNN-LSTM for GSL, we used temporal data taken from a 5 × 6 metal oxide semiconductor (MOX) gas sensor array, spaced 1.5 m apart, and an anemometer placed in the center of the sensor array in an open area outdoors. The output of the CNN-LSTM is one of thirty cells approximating the location of a gas source. We show that by using CNN-LSTM, we were able to determine the location of a gas source from sequential data. In addition, we compared several artificial neural network (ANN) architectures as well as trained them without wind vector data to estimate the complexity of the task. We found that ANN is a promising prospect for GSL tasks.

## 1. Introduction

Gas source localization (GSL) is the task of determining the place of origin from which gas is being released. It is an important research theme for those concerned with the emission of harmful gases. Landfill sites, mines, and factories are some of the areas likely to release harmful gases. Landfill sites regularly monitor for emission of greenhouse gases (GHGs), such as methane and carbon dioxide, using several personnel [1]; mines are areas known for occupational hazards such as being exposed to lethal gas or gas explosions [2]; and factories regularly monitor for leakages. Mines and factories use stationary gas sensors for detection but do not have the means for source localization. It is important that gas sources are located as soon as leaks are detected to prevent further damage from occurring. In all the above scenarios, the task of autonomous GSL can be applied.

In order for GSL to be successful, we first need to have a gas sensor that can detect the presence of a target gas in air. We also need an appropriate strategy to decide where the measurements should be made as well as effective algorithms for processing the gas sensor signal and estimating the source location [3]. The focus of this paper is on the processing of signals obtained from gas sensors at multiple locations in a given environment to estimate the location of the gas emission point. GSL can be extremely challenging even if gas sensors that can accurately and selectively measure the concentration of a target gas are developed. Since molecular diffusion is a slow process, the released gas is spread in the environment mainly by airflow. As the flow we encounter is almost always turbulent, a gas distribution with a complicated and constantly fluctuating shape is generated [3]. GSL algorithms have to be able to cope with the fluctuations caused by turbulence.

Nevertheless, autonomous GSL is useful for tasks that can be too laborious, repetitive, or hazardous for humans to perform [3]. Presently, there are two main methods for autonomous localization: using mobile robots or an array of stationary sensors. Mobile robots are easier to deploy than stationary sensors but the data collected is limited in comparison. Although deploying stationary sensors can be very time consuming, they provide richer information about the environment and can be kept on site if data must be collected regularly.

Research on using mobile robots for GSL first started by using bio-inspired gas tracking strategies, such as the strategies underlying the chemo-orientation behavior of bacteria and moths [4]. For example, male moths find their mates by tracking aerial trails of sexual pheromone released by conspecific females. The underlying strategy was found to be odor-gated anemotaxis, i.e., to fly upwind as long as contact with an aerial pheromone trail is maintained [5]. This strategy was implemented into mobile robots and tested to see if they were able to reach the gas source by tracking spatial gas distributions. They have been successful in environments where the distribution of gas is consistent [6,7] but suffer in outdoor environments because highly fluctuating wind often makes the gas distribution sporadic. Since then, researchers have explored strategies, such as the particle filter-based algorithm [8,9], that estimate the location of a gas source from signals obtained from sensors on a mobile robot. Therefore, GSL using mobile robots has a nature similar to methods using stationary sensors, as both try to estimate a gas-source location from gas concentration and wind velocity measured at multiple positions. In the methods proposed in [8,9], the gas source location is estimated by using a time sequence of the wind velocity vector to trace back the trajectory of gas puffs detected by a mobile robot to a common origin.

In this research, we focus on the use of an array of stationary sensors (sensor network). There has been research on using these sensor networks for GSL by calculating the position of a continuous gas source from the advection-diffusion model [10,11]. These methods use the Gaussian model to describe the gas concentration distribution in a turbulent plume, and estimate the source location by fitting the model equation to the measured concentration distribution. Another method that has been used is by using machine learning such as support vector machines (SVMs) and kernel ridge regression [12]. We instead propose the use of artificial neural networks (ANNs), specifically convolutional long-short term memory neural networks (CNN-LSTMs) [13,14,15], to model and estimate the location of a gas source in outdoor environments. We also hope that the insights provided by this research can be expanded into research using mobile robots.

We intend to deal with the ongoing problem of GSL in outdoor environments. Studies so far are usually limited to using simulations or environments of specific conditions [12,16]. This is because airflow in outdoor environments is usually intermittent and unpredictable making it difficult to model. Given enough data, we can train the CNN-LSTM to determine the type of environment and model necessary for GSL tasks. CNN-LSTMs have been successful for many visual learning tasks such as video description and activity recognition [13,14,15]. Using sequences of images (e.g., videos) as the input, the network can describe in detail the activity taking place in a scene. The time series data of a gas sensor network can be used in place of a sequence of images to describe the conditions of the environment and determine the location of a gas source.

In the work described in this paper, CNN-LSTM was tasked to estimate the location of a gas source from sequential data collected from an evenly distributed wired sensor array and an anemometer. The network assumes that a single high concentration gas source exists and sufficiently large responses are obtained. We also assume that the gas is released at a constant rate. Ethanol was used as the target gas in our experiments but future work will include methane gas detection. For practical applications, the work should be combined with gas discrimination techniques [17]. A single anemometer was used under the assumption that the direction of airflow is approximately uniform in large open areas [8]. CNN-LSTM is used in place of other ANN architectures because of its ability to extract information both spatially and temporally. This is because the CNN-LSTM leverages the combined strengths of convolutional neural networks (CNNs) and long short-term memory neural networks (LSTMs). CNNs have demonstrated outstanding performance in visual classification tasks [18]. LSTM on the other hand is a special type of recurrent neural network (RNN), which excels at tasks with sequential data. LSTMs stand apart from other RNN architectures for their ability to learn long-term dependencies and remove features [19,20]. By combining the strengths of CNN and LSTM architectures, CNN-LSTMs become a perfect choice for our task, simply because GSL tasks have spatio-temporal dependencies.

The remainder of this paper is organized into sections as follows: Section 2 introduces the materials and experiments conducted to collect the data necessary for training and evaluating ANNs as well as the methodology of using CNN-LSTM for GSL. Section 3 covers the results of training and evaluation as well as how it is interpreted. The final section summarizes the analysis and includes plans for further research.

## 2. Materials and Methods

### 2.1. Experimental Data

**Figure 1:**
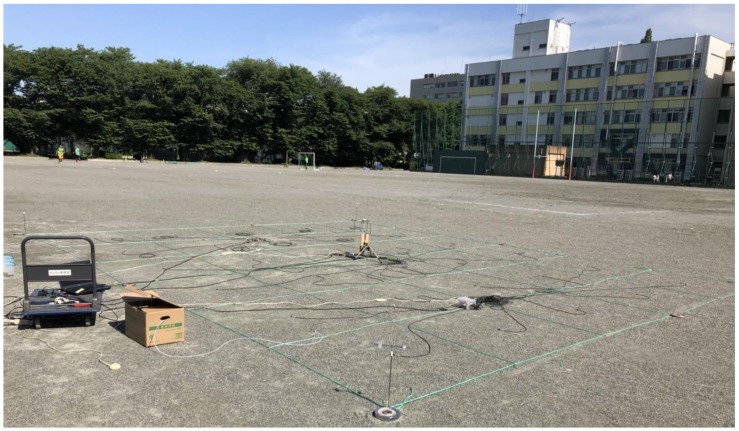
Experimental setup for obtaining training and validation data. MOX gas sensors are placed in each cell marked by the green strings. The anemometer can be visibly seen in the center.

To train the CNN-LSTM for GSL, sufficient training and validation data must be collected. We conducted experiments in an outdoor environment with a large open space that allowed wind from any direction. The experiments took place in the sports field of Tokyo University of Agriculture and Technology, shown in Figure 1, during the summer of 2018.

**Figure 2:**
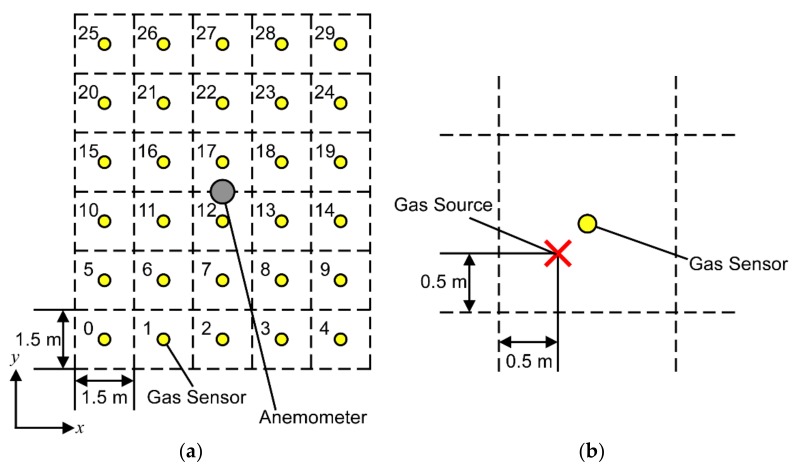
Schematic of experimental setup. (**a**) The numbers in each cell correspond to the location of a gas sensor; (**b**) Placement of gas source in each cell. The gas source was offset slightly between the sensor and the bottom-left corner of the cell.

We used an array of thirty commercially available metal oxide (MOX) gas sensors (TGS2620, Figaro Engineering Inc., Minoh, Japan) and one ultrasonic anemometer (Model 81000, R.M. Young Co., Traverse City, MI, USA). The gas sensors use tin dioxide for organic solvent detection and also respond to a variety of flammable gases. The gas sensors were spaced 1.5 m apart and arranged into a 5 × 6 matrix as shown in Figure 2a. Each cell in the matrix contains one gas sensor in the center. The anemometer is placed in the center of the entire matrix; a single anemometer was used under the assumption that the direction of airflow is approximately uniform in large open areas. The gas source used in the experiments was ethanol vapor with a flow rate of 500 mL/min and positioned in one of the cells as shown in Figure 2b. A porous filter was attached to the outlet so that vapor is released isotropically. Nearly saturated concentrations of ethanol vapor were generated by bubbling liquid ethanol. To obtain data sufficient for training, we would need to conduct experiments thirty times—one for each gas source location. Instead, we conducted experiments nine times to obtain data for each gas source in the cells numbered 〈0,1,2,5,6,7,10,12,18〉 in Figure 2a. The experiments were conducted over a span of four days. We obtained data for 〈6,12,18〉 on the first day, 〈0,1,2〉 on the third day, and 〈5,7,10〉 on the fourth day. On the second day, we obtained data for 〈6,12,18〉 again to evaluate the network with untrained conditions. We then rotated the data to obtain data for the remaining gas source locations. Data was logged for thirty minutes at a sampling period of ∆t=0.5 s, giving us a total of 3600 samples per gas source location. Each set of samples was divided into 300 time-step long sequences giving us a total of twelve datasets per gas source location. The datasets for each gas source location were then divided equally so that the first half of the sequences were used for training and the second halves were used for validation.

**Figure 3:**
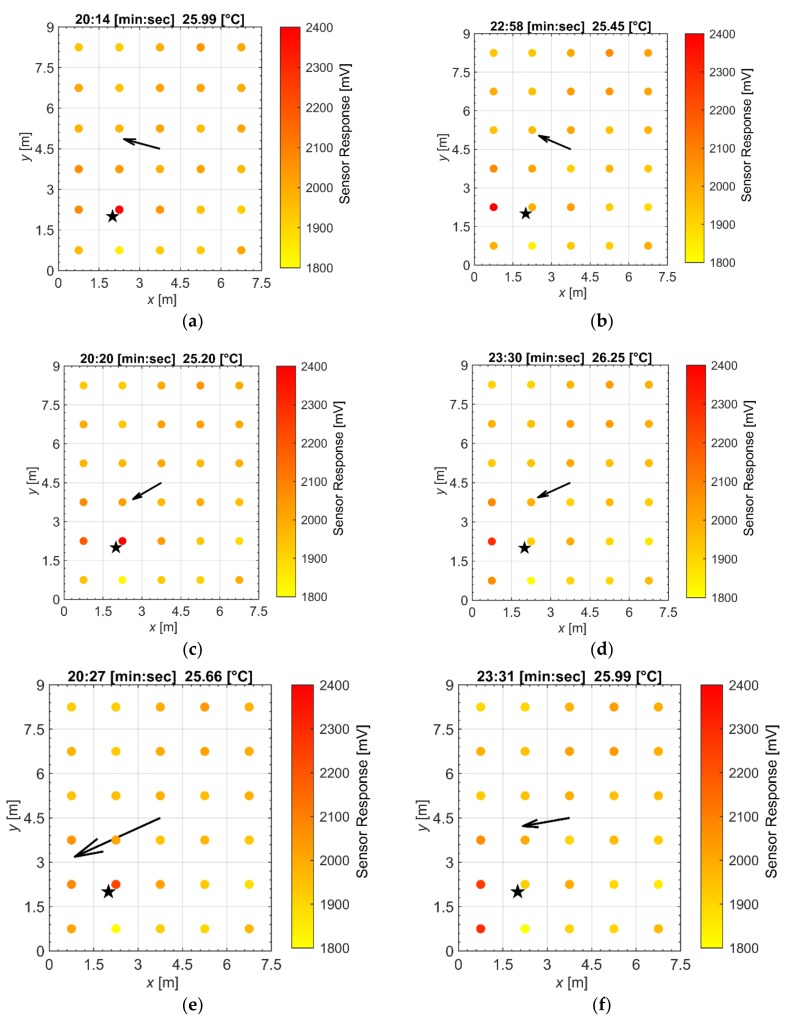
Frames of time-step data taken from two datasets of a gas source indicated by the star. The colors of the circles in each cell represent gas sensor data and the arrow indicates the magnitude and direction of the wind. (**a**–**c**) is one dataset to show that the max sensor response was located in the same cell as the gas source. (**d**–**f**) is another dataset of the same gas source; we can see that the max sensor response does vary depending on the wind.

**Figure 4:**
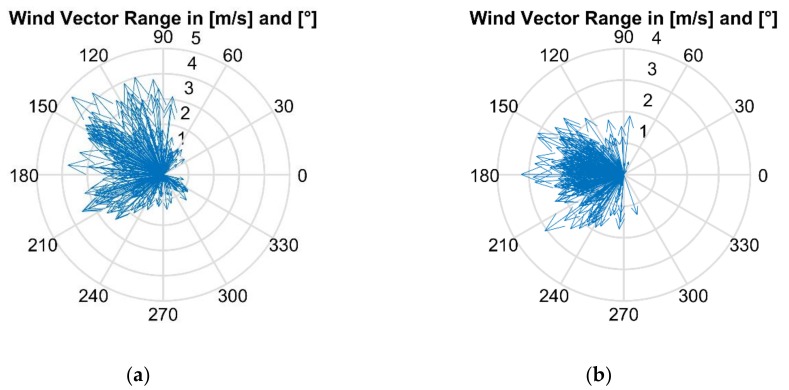
The wind vectors of two datasets are shown in polar coordinates to show the range. (**a**) The wind vector range of the dataset shown in Figure 3a–c. (**b**) The wind vector range of the dataset shown in Figure 3d–f.

To examine the spatial and temporal patterns learned by the ANNs, videos were created from the data using MATLAB (MathWorks Inc., Natick, MA, USA). Snapshots of two datasets are shown in Figure 3 with their respective wind spectrums shown in Figure 4. The wind angle and 2D velocity ranged from 36.4° to 346.8° and 0.16 m/s to 4.75 m/s, respectively. The wind velocity in the *z*-axis (the depth) ranged from −1.31 m/s to 1.07 m/s with averages across all datasets ranging from −0.07 m/s to 0.29 m/s. Since the density of ethanol vapor in the ppm concentration range detected by the gas sensors is similar to air (except for the vicinity of the gas source) and wind velocity in the *z*-axis was small, we assumed that gas released was mostly spread in 2D. Future work will include evaluation in 3D when dealing with lower density gases, e.g., methane. The resistance change of each gas sensor was converted into voltage change using a voltage divider circuit [1]. This voltage value increases as the gas concentration in the surrounding air becomes higher and was recorded together with the anemometer. Although we use voltage values, ANNs can accept any type of scalar input. Figure 3a–c illustrates that the max sensor response was always located in the same cell as the gas source and Figure 3d–f shows that the max sensor response was not; in addition, the sensor response pattern changes according to wind direction. 

However, there appears to be a delay to the change as shown in Figure 3e–f. In Figure 3e, the wind direction points slightly towards the bottom left corner cell but gas does not arrive at that cell until the time-step in Figure 3f. This delay was expected because of the time it takes for gas to travel in response to changes in the wind direction. We can also see in Figure 3f that gas appears to remain at the cell above the bottom left corner cell because of the slow recovery of MOX gas sensors. All datasets have a mix of these patterns which ensures that the ANNs do not simply learn from the location of the max sensor response. We checked the videos and confirmed that large resistance changes are correlated with the source location and wind direction, suggesting the small influence of wind on the sensors’ response.

### 2.2. Methodology

**Figure 5:**
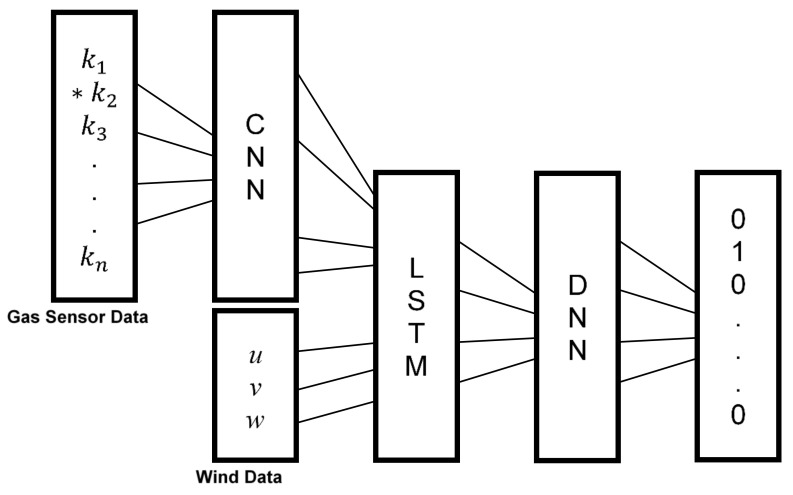
Simplified architecture of the CNN-LSTM for GSL. The output of the CNN-LSTM is an array of binary values where 1 represents the location of the gas source. The output in this figure shows that the gas source is in the same cell as $k_{2}$.

We propose CNN-LSTM for GSL because of its ability to extract features that is both spatially and temporally deep. CNN-LSTMs are used for many visual learning tasks but are also known to be used for speech recognition and natural language processing. We classify GSL as a visual learning task, specifically classification from a sequence of images. This is because the gas sensor array data $\langle k_{1},k_{2},\ldots ,k_{n}\rangle$, where k is the sensor response and n is the sensor position, can be arranged to resemble a sequence of monochrome images. Each “image” is a 5 × 6 matrix $r_{t}$ where t is the time-step of a fixed length sequence and $\langle k_{1},k_{2},\ldots ,k_{n}\rangle \in r_{t}$. The architecture of our CNN-LSTM is shown in Figure 5. At each time-step, $r_{t}$ is processed by the CNN layer [18]. The CNN layer consists of one convolutional layer followed by a hyperbolic tangent activation function and two sets of a fully connected layer and rectified linear unit (ReLU) activation function. The output is often written as:

(1) $$\Phi \left(r_{t}\right)=p\left(Wr_{t}+b\right),$$

where p is an activation function, W is the weight matrix and b is the bias. The kernel shape of the feature transformation function in the convolutional layer was set to 〈3,3,5〉 with no padding. The purpose of the CNN layer is to process each input $r_{t}$ into a feature transformation function to extract spatial features.

Once the last input in the sequence has been processed, the outputs $\langle \Phi \left(r_{1}\right),\Phi \left(r_{2}\right),\ldots ,\Phi (r_{T})\rangle$ of length T and the wind vectors $\langle U_{1},U_{2},\ldots ,U_{T}\rangle$ are processed by the LSTM layer. Here, the LSTM layer learns the temporal features. Typical RNNs have difficulty learning long-term dependencies because of exploding or vanishing gradients resulting from gradients propagating at each time-step. LSTMs were developed as a solution by using structures called gates. These gates regulate information or memory in the LSTM called the cell state $C_{t}$ by removing or adding information every time-step depending on the previous LSTM output $h_{t-1}$ and the current input $\Phi \langle (r_{t}),U_{t}\rangle$. The action of removing information is synonymous to “forgetting” and is one of the defining features of LSTMs. This is advantageous for GSL tasks where wind is highly intermittent with frequent periods of stagnation. The data taken during these periods of stagnation would be discarded by the LSTM, simplifying its task of learning the temporal dynamics. At each time-step the LSTM updates the following:

(2) $$f_{t}=\sigma \left(W_{f}\cdot \left[h_{t-1},\Phi \left(r_{t}\right),U_{t}\right]+b_{f}\right),$$

(3) $$i_{t}=\sigma \left(W_{i}\cdot \left[h_{t-1},\Phi \left(r_{t}\right),U_{t}\right]+b_{i}\right),$$

(4) $$C\prime_{t}=\tanh\left(W_{C}\cdot \left[h_{t-1},\Phi \left(r_{t}\right),U_{t}\right]+b_{C}\right),$$

(5) $$C_{t}=f_{t}*C_{t-1}+i_{t}*C\prime_{t},$$

(6) $$o_{t}=\sigma \left(W_{o}\cdot \left[h_{t-1},\Phi \left(r_{t}\right),U_{t}\right]+b_{o}\right),$$

(7) $$h_{t}=o_{t}*\tanh\left(C_{t}\right),$$

where $f_{t}$ is the forget gate, $i_{t}$ is the input gate, $C\prime_{t}$ is a vector of new candidate values for the cell state $C_{t}$ and $o_{t}$ is the output gate. “*” denotes the element-wise product of vectors. From (5), we can see that the cell state is updated by discarding values from the previous cell state $C_{t-1}$ decided by the forget gate and adding new values $C\prime_{t}$ decided by the input gate. The output of the LSTM $h_{t}$ is called the hidden state which is the cell state filtered by the output gate. The cell state contains pertinent information about what should be output by the LSTM. For GSL tasks, if the LSTM determined that the location of the gas source is at $y_{n}$ where n is the n-th sensor location then the cell state may contain information about all sensors and the output gate filters for only the nearest sensor locations around $y_{n}$. 

Since wind vector data is also included in the input of the LSTM, the cell state may contain information about how these sensors should respond should the wind vector change at the next time-step. The outputs of the LSTM $\langle h_{1},h_{2},\ldots ,h_{T}\rangle$ are then processed by the deep neural network (DNN) layer which consists of two sets of a fully connected layer and ReLU activation function and one set of a fully connected layer and sigmoid activation function. The sigmoid function squashes the output between 0 and 1 with each output being independent of each other, this allows us to train the network for multiple sources in the future. In the case presented in this paper, a 300 time-step long sequence measured over 150 s is given to the network. The per time-step predictions of the LSTM layer are merged for the whole sequence and processed by the DNN layer to classify them as one of thirty labels representing the location of the gas source. Therefore, one estimate of the gas source location is provided after processing the 150 s long sensor data. We chose 150 s considering the recovery time of the gas sensors (~30 s) and the time scales of the environment (we observed changes in the wind direction over several tens of seconds).

### 2.3. Optimizations

To reduce the significance of weight initialization and prevent overfitting during training, batch normalization and dropout layers were added to the CNN and DNN layers. The purpose of batch normalization is to not only speed up training but to reduce the significance of weight initialization [21]. The purpose of dropout is to reduce overfitting [22], the ratios were all set to 0.5 to essentially reduce the number of nodes connected to the next layer by half after each dropout. It is also important to note that the number of outputs for each fully connected layer can be controlled, which directly affects the total number of parameters in the network and the speed of training. The number of outputs of all fully connected layers in the CNN and DNN layers were set to 300 unless they are followed by the last activation function, in which case the number of outputs was set to 30 to match the number of labels. The fully connected layers in the LSTM layer affect the size of the cell state. In this case, we set the number of outputs of these layers to 33 to match the number of inputs at each time-step. These optimizations are also used in other network architectures for fair comparison.

### 2.4. Training and Validation

In addition to training the CNN-LSTM, we have trained several network architectures for comparison. We have also trained the networks using only gas sensor data to see how removing wind vector data affects the results. The network architectures used for comparison are LSTM, CNN-DNN, and DNN. Each architecture is simply the CNN-LSTM split into their main components to estimate the complexity of the GSL task. The program used for training and validation was Neural Network Console (Sony Network Communications Inc., Tokyo, Japan). Since training and validation data is limited, we used an adaptive optimizer which gives good results when using input data that is sparse. The adaptive optimizer used was the adaptive momentum (Adam) optimizer with default parameters. Each network was trained using the training datasets, comprised of six datasets per gas source location, and considered converged when validation error no longer improved after several epochs. The networks were then evaluated using the validation datasets which also comprise of six datasets per gas source location; Neural Network Console provides us performance data necessary for comparison. We used binary cross entropy as the loss function for training and validation error.

## 3. Results and Discussion

In this section, we evaluate the performance of each network using standard performance metrics for classification problems in machine learning: accuracy, precision, recall and F1-score. Accuracy is the measure of the number of correct predictions over the total predictions made. There are exactly thirty labels with six input sequences for each label, making the metric valid because of balanced data. Precision is the measure of true positives over the sum of true positives and false positives. Recall is the measure of true positives over the sum of true positives and false negatives. Precision and recall are measurements used to determine the proportion of false positives and false negatives respectively. If we are concerned about both proportions, F1-score is used as a single measurement. The results of evaluating the networks are shown in Table 1 and Table 2. Table 1 compares the performance of each network architecture using all input data (gas sensor and wind vector data) and Table 2 shows us the comparison using only gas sensor data. The validation error and epoch in the tables show when the lowest validation error was achieved before convergence, where an epoch is one pass of all training datasets to the networks.

If we simply chose the place with the maximum average gas sensor response as the estimated gas source location, the accuracy was only 45.6%. Much higher accuracy was obtained for all ANNs tested. The results in Table 1 show that CNN-LSTM performed the best in all metrics (accuracy, precision, recall, and F1-score) while LSTM performed the worst. The LSTM architecture may have suffered because inputs were directly injected into the LSTM layer. This is because when spatial features are extracted from the CNN layer of the CNN-LSTM, we are essentially filtering the data for the LSTM layer to process. We also see that the LSTM and CNN-DNN architectures show fair improvements when wind vector data was removed from the input. This could be due to the delay between the gas sensor response and change in wind direction being removed from the network. Although we see that CNN-LSTM performed the best when compared to the other networks, the difference is modest. This tells us that the difficulty of the task was simpler than anticipated. It could also be due to the size of the sensor array and distance between sensors, reducing the number of sensors or increasing the distance between them may show more disparity between network architectures. While the non-recurrent networks performed just as well, they will not be considered for further research because of their inability to recognize temporal patterns, using data from untrained weather conditions may make them unable to predict.

**Figure 6:**
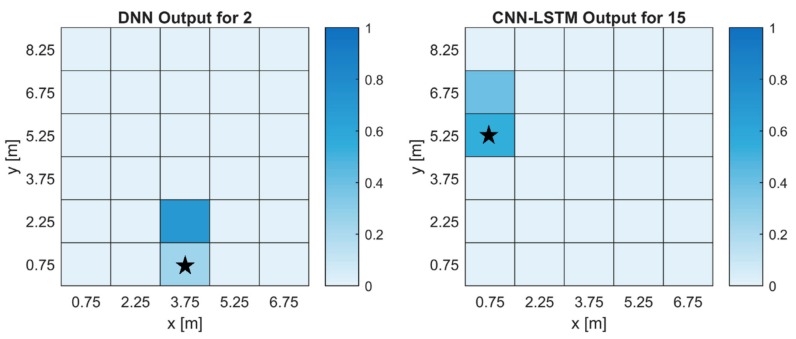
Heatmaps of the ANN output with the lowest prediction rate for each ANN architecture. The labels are the same as the cell numbers in Figure 2. The true gas source location is indicated by the star. (**a**) The output of a label with the lowest prediction rate for the DNN. (**b**) The output of a label with the lowest prediction rate for the CNN-LSTM.

**Figure 7:**
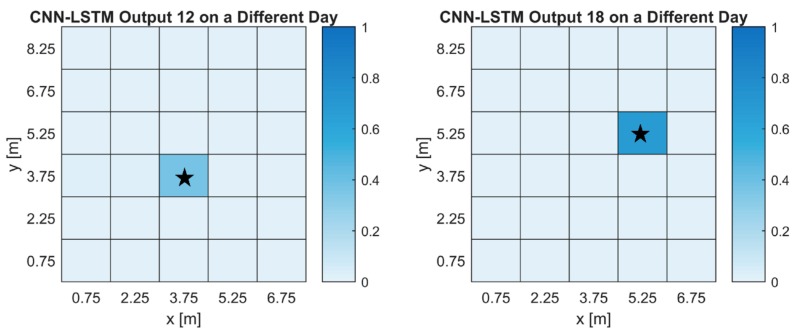
Heatmaps of the CNN-LSTM output using data collected on a different day. The labels are the same as the cell numbers in Figure 2. The true gas source location is indicated by the star. (**a**) The output of the CNN-LSTM for a gas source placed in cell 12. (**b**) The output of the CNN-LSTM for a gas source placed near cell 18.

For the CNN-LSTM, there were 26 labels with a prediction rate of 6/6, two labels with a prediction rate of 3/6 and two labels with 4/6 and 5/6. The DNN had 22 labels with a prediction rate of 6/6, four labels with a prediction rate of 5/6, two labels with 4/6 and two labels with 2/6. To get a better understanding of what the networks output, heatmaps were created from labels with the lowest prediction rate and are shown in Figure 6. Figure 6a shows us the average output of a gas source for the DNN architecture while Figure 6b shows us the average output of a gas source for the CNN-LSTM architecture. The first thing we notice is that for both architectures, prediction values are highest around the true gas source location. This is a good indication that the networks are learning about the gas distribution. In Figure 6a we can see that while the true gas location had low prediction values, the location above was high. For our purposes, this can still be an acceptable outcome since we narrow our search near the true location. To show the practicality of using ANNs for GSL, we have evaluated the CNN-LSTM network using data collected on a different day, shown in Figure 7. The heatmaps show the average output for two of the three gas source locations. We can see that the outputs overlap with the true source location. Since evaluating networks with untrained conditions, in this case weather, can produce substantially lower prediction rates, these results showed that as long as weather conditions are somewhat similar, adequate predictions can be made. It also shows that the networks are not overfitted. The CNN-LSTM predicted 15 of the 18 datasets collected.

## 4. Conclusions

In this research, the CNN-LSTM was proposed to model and estimate the location of a gas source in outdoor environments using time series data of a gas sensor network and anemometer as the input. The data was obtained through several experiments in large open areas for practicality. Through training and evaluation, we found that the CNN-LSTM was able to successfully predict the location of a gas source. We also evaluated several networks for comparison as well as removed the wind vector data from the input to evaluate the difficulty of the task given to the networks. The results show that the task of determining the location of a single gas source was simpler than we believed. This is partly because the size of the sensor array and the distance between sensors gave us relatively high-resolution data—decreasing the number of sensors or increasing the distance between sensors could affect the results. We believe the results of this research make ANNs a promising prospect for GSL tasks and will be further studied. For future research, we plan to use CNN-LSTM to estimate the location of multiple gas sources with their respective emission rates. We will also collect data under different weather conditions and reduce the number of sensors or length of the input sequence to see how the results will change, we will give the network a portion of the data each time-step to imitate the movement of a mobile robot. Although the proposed ANNs can be applied to different types of gas sensors and target gases, future work also needs to address the effect of the density mismatch between the target gas and ambient air on estimating the gas source location.

## Figures and Tables

**Table 1:** Performance metrics of each ANN when wind vector data is included as an input.

Model	Accuracy	Precision	Recall	F1-Score	Validation Error	Epoch
CNN-LSTM	95.0%	96.5%	95.0%	94.7%	0.0115	160
LSTM	85.0%	87.3%	85.0%	84.9%	0.0307	310
CNN-DNN	90.0%	92.7%	90.0%	89.1%	0.0273	260
DNN	91.1%	93.0%	91.1%	90.6%	0.0200	350

**Table 2:** Performance metrics of each ANN when wind vector data is removed.

Model	Accuracy	Precision	Recall	F1-Score	Validation Error	Epoch
CNN-LSTM	93.9%	95.6%	93.9%	93.6%	0.0116	300
LSTM	88.9%	89.9%	88.9%	88.4%	0.0214	290
CNN-DNN	93.3%	94.8%	93.3%	93.0%	0.0135	300
DNN	88.3%	91.6%	88.3%	87.6%	0.0206	270
